# Targeting strategies with lipid vectors for nucleic acid supplementation therapy in Fabry disease: a systematic review

**DOI:** 10.1007/s13346-024-01583-0

**Published:** 2024-04-08

**Authors:** Julen Rodríguez-Castejón, Marina Beraza-Millor, María Ángeles Solinís, Alicia Rodríguez-Gascón, Ana del Pozo-Rodríguez

**Affiliations:** 1 Pharmacokinetic, Nanotechnology and Gene Therapy Group (PharmaNanoGene), Faculty of Pharmacy, Centro de Investigación Lascaray Ikergunea, University of the Basque Country, UPV/EHU, Paseo de la Universidad 7, Vitoria-Gasteiz, 01006 Spain; 2 Bioaraba, Microbiology, Infectious Disease, Antimicrobial Agents and Gene Therapy, Vitoria-Gasteiz, 01006 Spain

**Keywords:** Fabry disease, Targeting, Nucleic acid, Lipid nanoparticles, PRISMA

## Abstract

Fabry disease (FD) results from a lack of activity of the lysosomal enzyme α-Galactosidase A (α-Gal A), leading to the accumulation of glycosphingolipids in several different cell types. Protein supplementation by pDNA or mRNA delivery presents a promising strategy to tackle the underlying genetic defect in FD. Protein-coding nucleic acids in FD can be either delivered to the most affected sites by the disease, including heart, kidney and brain, or to specialized organs that can act as a production factory of the enzyme, such as the liver. Lipid-based systems are currently at the top of the ranking of non-viral nucleic acid delivery systems, and their versatility allows the linking to the surface of a wide range of molecules to control their biodistribution after intravenous administration. This systematic review follows the Preferred Reporting Items for Systematic Reviews and Meta-Analysis (PRISMA) statement guidelines and provides an overview and discussion of the targeting ligands that have been employed so far to actively vectorize intravenously administered non-viral vectors based on lipid carriers to clinically relevant organs in the treatment of FD, for protein-coding nucleic acid (pDNA and mRNA) supplementation. Among the thirty-two studies included, the majority focus on targeting the liver and brain. The targeting of the heart has been reported to a lesser degree, whereas no articles addressing kidney-targeting have been recorded. Although a great effort has been made to develop organ-specific nucleic acid delivery systems, the design of active-targeted carriers with high quality, good clinical translation, and large-scale manufacturing capacity is still challenging.

**Graphical Abstract:**

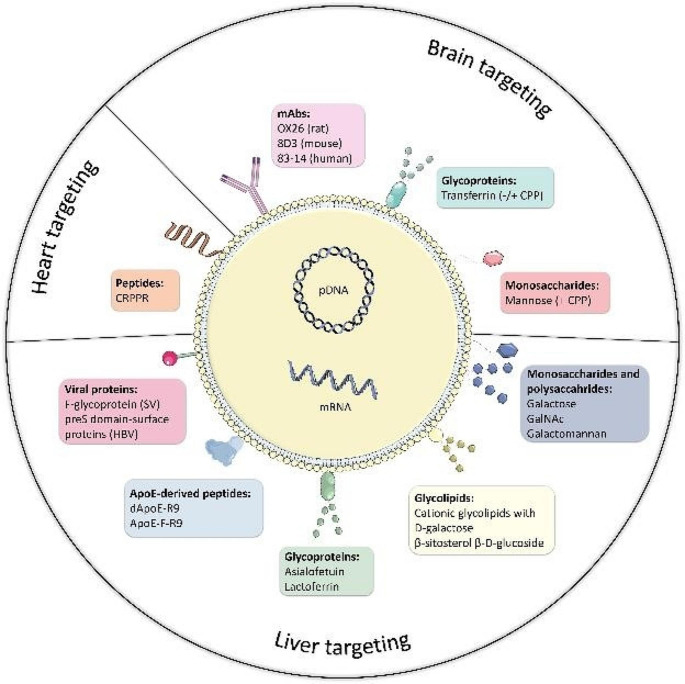

**Supplementary Information:**

The online version contains supplementary material available at 10.1007/s13346-024-01583-0.

## Introduction

Fabry disease (FD, OMIM # 301,500) is a devastating, progressive genetic disease caused by pathogenic variants in the *GLA* gene (Xq21.3-q22), which encodes the lysosomal enzyme α-Galactosidase A (α-Gal A). Deficiency in α-Gal A activity leads to systemic accumulation of glycosphingolipids, predominantly globotriaosylceramide (Gb3) and its deacylated derivative globotriaosylsphingosine (lyso-Gb3), within the lysosomes of multiple cells types, including endothelial, vascular, smooth muscle, renal, cardiac and nervous cells [1, 2]. Gb3 accumulation in the intracellular compartment is associated with structural damage and loss of function in different tissues; the organs that are mainly affected by the disease are the heart, kidney and nervous system [2, 3]. Consequently, end-stage renal disease, heart dysfunction (e.g., hypertrophic cardiomyopathy, cardiac arrhythmias, valvular disease) and cerebrovascular events (e.g., transient ischemic attacks, ischemic strokes) are the major life-threatening disease manifestations [4].

Current available treatment options for FD include intravenous (i.v.) enzyme replacement therapy (ERT) with recombinant enzymes (agalsidase α (Replagal^®^) and agalsidase β (Fabrazyme^®^)) and oral chaperone therapy (Migalastat (Galafold^®^)). Although existing therapies have shown to improve the overall quality of life of patients, they exhibit important limitations. Regarding ERT, efficacy is greatly conditioned by the initiation age, approved enzymes have low tissue penetration and none of them crosses the blood–brain barrier (BBB), administration causes infusion associated reactions and may induce the production of anti-drug antibodies with neutralizing effect, requires lifetime i.v. infusion every 2 weeks and it involves a high cost [5]. As for chaperon therapy, only patients with mutant forms amenable to Migalastat, mainly missense mutations that result in misfolded α-Gal A and premature degradation of the protein, are potential candidates for this treatment option [6].

These concerns with current treatments for FD have opened the way for developing new therapeutics, such as nucleic acid supplementation therapy approaches. That strategy applied to FD is based on the administration of nucleic acids encoding the α-Gal A enzyme, so that it is synthesized by patient’s native cells. Endogenously produced enzyme by gene supplementation undergoes natural translational and post-translational modifications, enhancing stability and reducing immunogenicity as compared to recombinant α-Gal A [7].

**Fig. 1:**
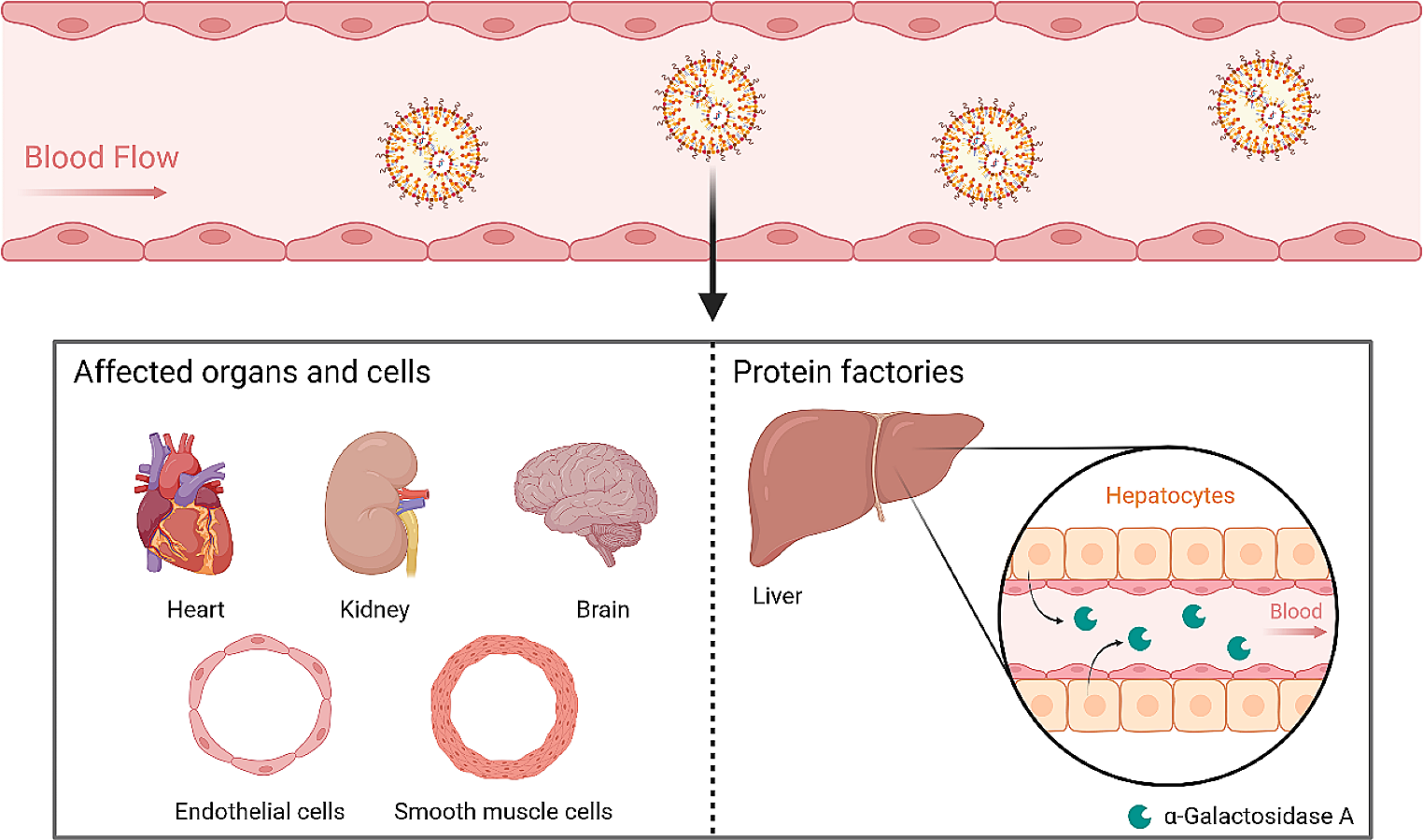
Target organs and cells for nucleic acid-based supplementation therapy in Fabry disease. Nucleic acid therapies can be designed to target the most affected organs and cells by the disease, or to target organs that can act as a production factory of the enzyme to be secreted and taken up by other cells. Created with BioRender.com

Gene therapies for FD can benefit from the cross-correction phenomenon, which enables the uptake of expressed and secreted α-Gal A by cells other than transfected ones. This eliminates the need to reach every individual affected cell and may confer long-term enzyme cross-correction depending on the cell type initially transduced [7, 8]. In this sense, nucleic acid therapies can be designed to target the most affected organs by the disease or to target a long lifespan specialized cell population, such as hepatocytes, as a production depot of the therapeutic protein for uptake by other cells [9, 10] (Fig. 1).

Success of nucleic acid-based therapies depends largely on the delivery system used, which must ensure protection of the cargo against degradation and facilitate its internalization and intracellular delivery into the target cells [11]. Considering the natural transduction properties of most viruses, viral vectors have been at the forefront of nucleic acid delivery systems. However, more recently, the design and development of non-viral vectors based on biocompatible materials has taken the lead in order to overcome the oncogenic and immunogenic risks of viral vectors [12], and the approval of SARS-CoV-2 messenger RNA (mRNA)-based vaccines has placed lipid-based vectors at the top of the ranking of non-viral nucleic acid delivery systems [13].

One of the most important obstacles to the use of non-viral vectors for systemic administration is the specific targeting to the desired tissue to avoid off-target effects and enhance the uptake and efficacy. Active targeting can be achieved by incorporating or attaching ligands on the surface of the carrier that specifically bind to receptors present exclusively in the target tissue or cell [14–16]. Different types of molecules have been employed as active targeting moieties of non-viral nucleic acid delivery systems, such as antibodies [17] or antibody fragments [18], peptides [19] and carbohydrates [20]. However, it remains a challenge to design targeted carriers with high quality and good clinical translation and large-scale manufacturing capacity [21].

This descriptive systematic review aims to provide an overview and discussion of the targeting ligands that have been employed so far to direct intravenously administered non-viral vectors based on lipid carriers, for protein-coding nucleic acid (plasmid DNA (pDNA) and mRNA) supplementation to clinically relevant organs in the treatment of FD. Target tissues include those that are most affected by the disease (heart, kidney, brain, smooth muscle and endothelial cells) or those that can act as a production factory of the enzyme, such as the liver. In addition, the challenges to be overcome for clinical translation of active targeted lipid-based nucleic acid supplementation therapy for FD, and future perspectives are highlighted.

## Methods

The methods to perform this systematic review follow the Preferred Reporting Items for Systematic Reviews and Meta-Analysis (PRISMA) statement guidelines [22].

### Search strategy and screening

According to Participants, Interventions, Control, and Outcomes (PICO) principles [23], the following focused question was set: “Which targeting ligands have been employed to deliver protein-coding nucleic acids (pDNA and mRNA) with lipid non-viral vectors to tissues/organs relevant in the treatment of FD?” The research question was conceived as follows: (P) participant: animals/humans intravenously administered; (I) intervention: targeted lipid non-viral nucleic acid delivery systems; (C) control group: non-targeted lipid non-viral nucleic acid delivery systems; (O) outcome: biodistribution profile and transfection efficacy.

To filter studies relevant to the focused question, manuscripts were searched in 3 electronic databases: PubMed, Web of Science (WoS) and Scopus. A search strategy was developed with keywords based on eligibility criteria, and search was conducted using the tittle and abstract headings. Full search strategies for all databases are presented in Table S1(Online Resource 1). The initial literature search was conducted in September 2023. In order to maximize the scope of the search, no specific start year was set for the search of the reports in the databases, encompassing the widest possible range of information. An updated search was conducted the 15th of November 2023 and included records were updated.

Three of the authors independently screened titles and abstracts of the manuscripts following the eligibility criteria (Table 1). One author retrieved full texts for eligibility. Three authors independently assessed eligible articles. Remaining uncertainties of studies to be considered for the review were discussed until a consensus was reached.

### Eligibility criteria

Inclusion and exclusion criteria for eligibility of the studies are summarized in Table 1.

**Table 1:** Eligibility criteria

	Inclusion criteria	Exclusion criteria
Target organs	Organs relevant in the treatment of FD: heart, kidneys, brain and liver (hepatocytes); smooth muscle and vascular endothelial cells	Any organ other than those included in the inclusion criteria; tumor targeting
Delivery system	Non-viral lipid nucleic acid delivery systems	Non-lipid nucleic acid delivery systems; viral vectors
Nucleic acid cargo	Protein-coding nucleic acids (pDNA and mRNA)	Any nucleic acids other than pDNA and mRNA; drugs or bioactives other than pDNA and mRNA
Type of study	In vivo studies	In vitro studies
Route of administration	Intravenous	Any route other than the intravenous
Type of report	Original research studies	Reviews, letters, editorial material, book chapters, proceeding papers, meeting abstracts, expert opinions, patents and dissertation/thesis
Language	Reports written in English	Reports written in a language other than English

FD: Fabry disease. mRNA: messenger RNA. pDNA: plasmid DNA

### Data extraction

Study characteristics were extracted by a single author and included the following parameters: target organ/tissue/cell, targeting moiety, target receptor, anchoring method of the ligand to the delivery system, type of lipid delivery system, nucleic acid cargo and experimental parameters related to biodistribution and gene expression.

### Quality assessment of the studies included

Assessment of methodological quality for each study followed the Animal Research: Reporting of In Vivo Experiments 2.0 (ARRIVE 2.0) guidelines [24]. Studies were scored using the recommended set of 21 items **Table S2** (Online Resource 1), as detailed by García-Gonzalez et al. [25]. If all subitems complied, it was indicated with “Reported (= 2 points)”, if all subitems did not comply, it was noted with “Unclear (1 = point)”, and if no reporting was done, it was indicated as “Not reported (= 0 point)”. Scores were summed to a total score. For each study, the total score was divided by the maximum score (42 points) to obtain a predefined quality coefficient (0.8–1: Excellent; 0.5–0.8: Average; < 0.5: Poor) [25, 26]. To assess the reporting of each item across studies, the percentage of studies that had reported, unclear, and not reported each individual item was calculated.

Assessments were done by three independent authors, and disagreements were pooled among the authors for discussions. Studies were not excluded based on this assessment, but its results were considered in the overall discussion.

## Results

### Identification and selection of studies

**Fig. 2:**
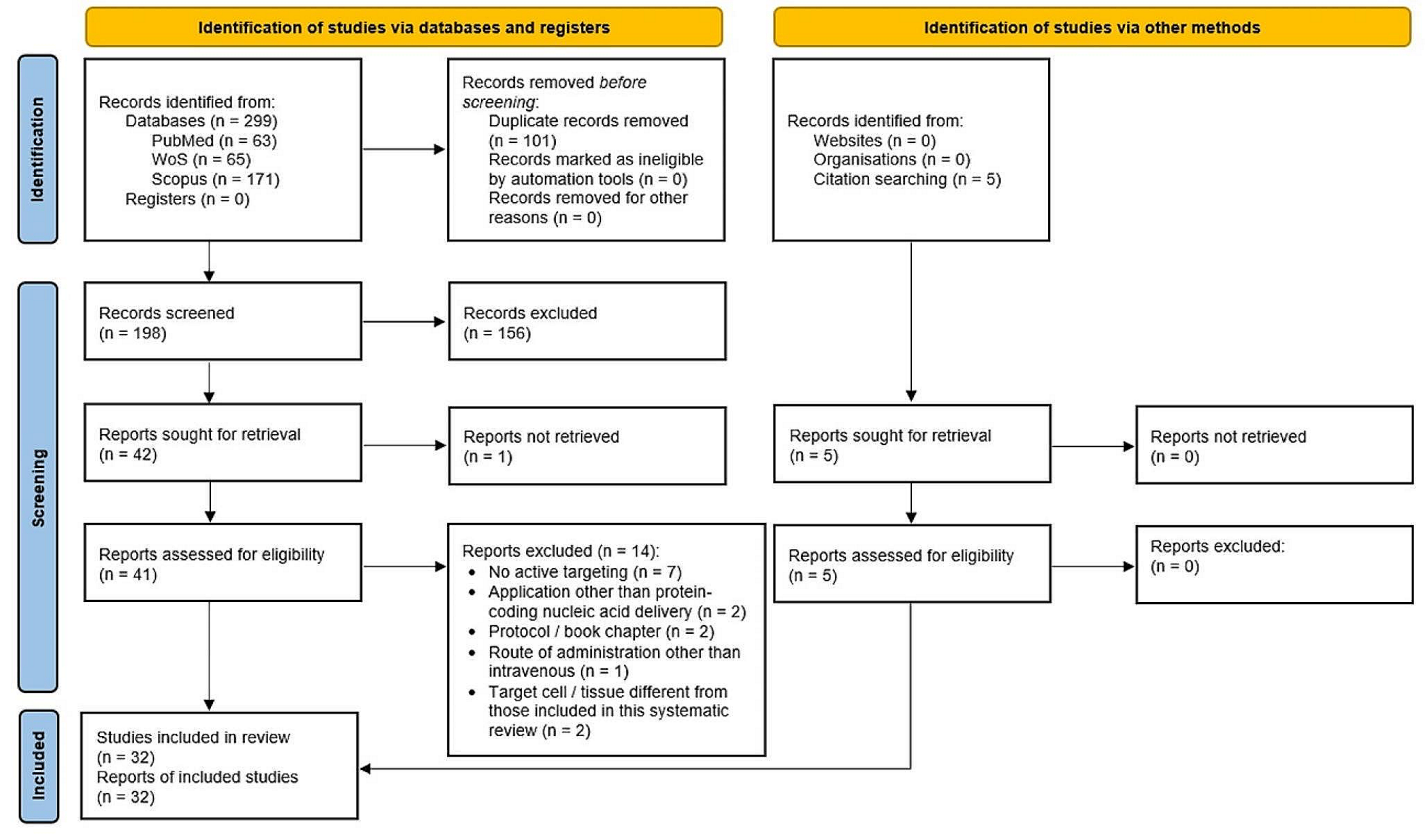
PRISMA 2020 flow diagram for new systematic reviews which included searches of databases, registers and other sources. WoS: Web of Science

After following the steps of the flow diagram included in Fig. 2 for the selection of studies, 32 reports were finally recorded for data extraction and analysis. Meta-analysis was not conducted due to the scarcity and heterogeneity of the studies. Thus, our systematic review is descriptive.

### Quality assessment of the studies included

**Fig. 3:**
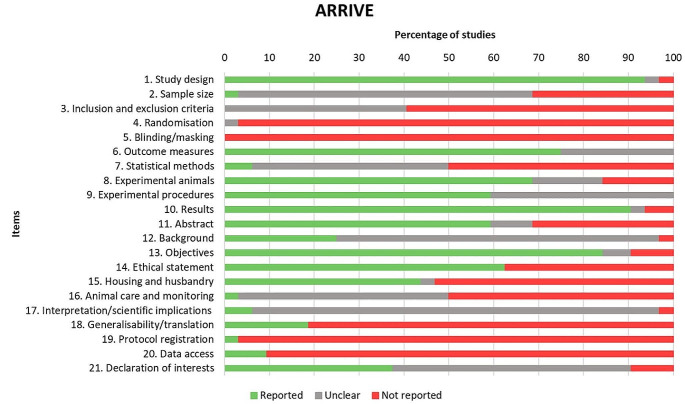
Quality assessment of the studies according to the 21 items of the ARRIVE 2.0 guidelines (Animal Research: Reporting of In Vivo Experiments 2.0)

Figure 3 represents the percentage frequencies of each item of the ARRIVE 2.0 guidelines. The most frequently reported items (> 80% of the studies reported it) were (1) study design, (10) results and (13) objectives. Conversely, items least frequently reported (> 80% of the studies did not report it) were (4) randomisation, (5) blinding/masking, (18) generalisability/translation, (19) protocol registration and (20) data access.

The mean study quality coefficient was 0.48. **Table S2** (Online Resource 1) shows the scores of each item of the ARRIVE 2.0 and the calculated coefficients for each study. Fifteen studies (47%) were rated as average (coefficient 0.5–0.8) and 17 (53%) as poor (coefficient < 0.5). None of the studies were rated as excellent (coefficient 0.8–1).

### The studies

**Fig. 4:**
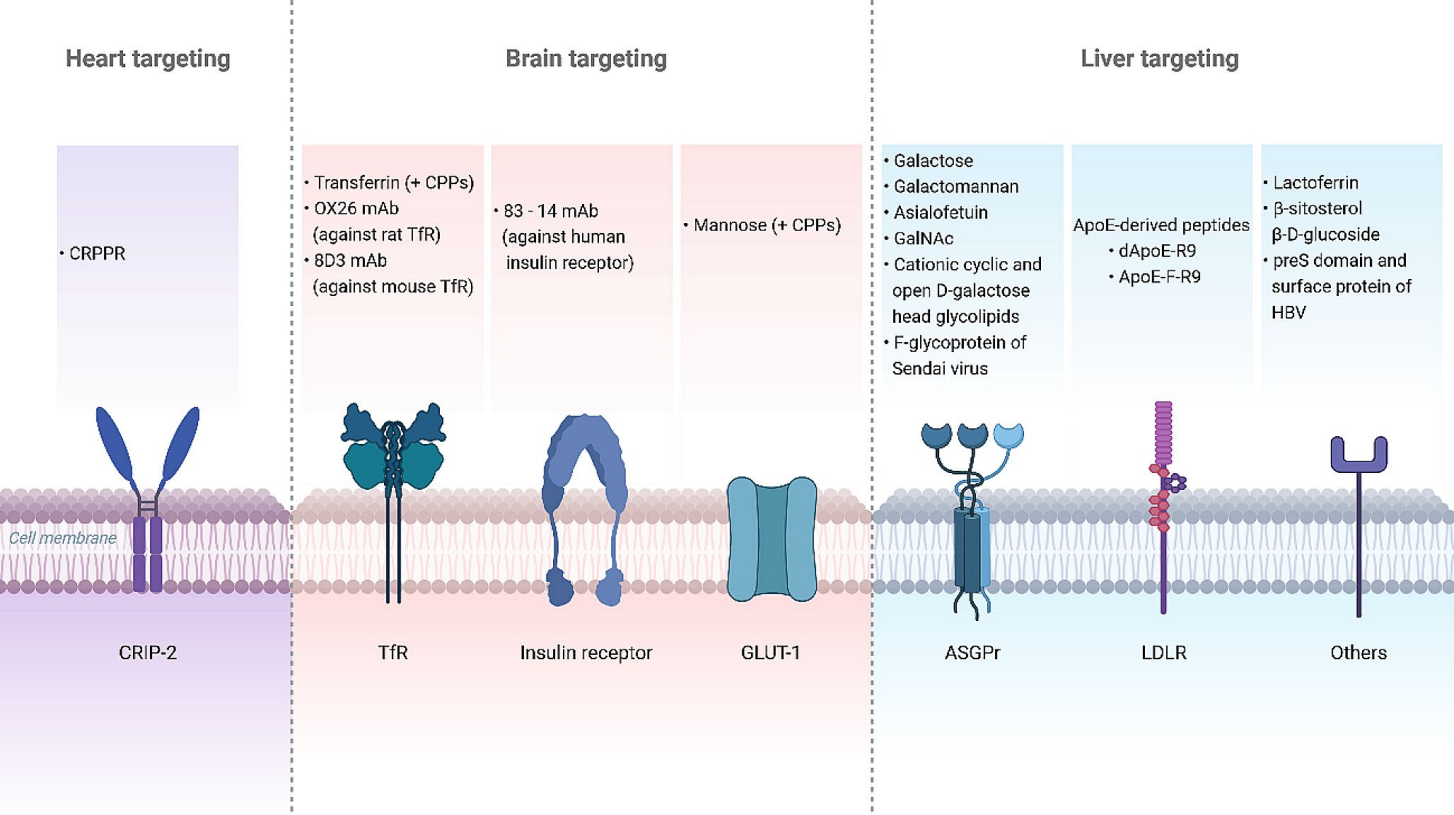
Target receptors and ligands identified for lipid-based pDNA and mRNA delivery to heart, brain and liver. ApoE: Apolipoprotein E. ASGPr: asialoglycoprotein receptor. CPP: cell-penetrating peptide. CRIP-2: cysteine-rich-protein-2. GalNAc: N-acetylgalactosamine. GLUT-1: glucose transporter-1. HBV: hepatitis B virus. LDLR: low-density lipoprotein receptor. mAb: monoclonal antibody. TfR: transferrin receptor. Cell penetrating peptides (CPP) comprise poly-L-arginine, penetratin, vascular endothelial-cadherin-derived peptide, pentapeptide QLPVM, HIV-1 trans-activating protein (TAT), melittin, Kaposi fibroblast growth factor (kFGF), penetration accelerating sequence–R8 or rabies virus glycoprotein. Created with BioRender.com

The most relevant information extracted from included studies is presented below classified according to the target organ they report. Additionally, **Table S3** (Online Resource 1) summarizes data extracted from included studies. Among the studies included in the review, 2 described heart targeting, 18 brain targeting and 12 liver targeting. No records targeting kidneys, smooth muscle cells or vascular endothelial cells were identified. As it is shown in **Table S3** (Online Resource 1), 30 reports used liposomes as delivery system, 1 used solid lipid nanoparticles and 1 hybrid lipid-polymer system. Regarding the cargo, except for 4 reports that described either unloaded or fluorescent dye-loaded carriers (those were included as they were designed for the delivery of nucleic acids), 1 study employed mRNA as nucleic acid, while the remaining 27 used pDNA. Figure 4 illustrates the target receptors and ligands identified in the search of the systematic review.

#### Heart targeting

CRPPR is a linear peptide containing arginine that has been identified as a heart-homing peptide owing to its ability to specifically bind to heart endothelium, and cysteine-rich protein-2 (CRIP-2) has been proposed as the receptor. Zhang H et al. [27] designed a radiolabeled liposome functionalized with CRPPR to bind to the heart. Using dynamic positron emission tomography in a mouse model, they investigated the biodistribution and pharmacokinetics. Their results revealed that CRPPR-liposomes accumulated in the heart in a concentration of 44% of injected dose per gram of tissue within 100 s, resulting in a value 9.4 fold greater than non-targeted liposomes. In another study [28], the same authors aimed to assess if the cargo of CRPPR-liposomes is internalized by endothelial cells and subsequently transported into tissue. They also evaluated the accumulation of the liposomes in models of cardiovascular disease. According to their findings, CRPPR-liposomes accumulated in both healthy and diseased hearts, with the cargo accumulating in the tissue within minutes and remaining detectable even after 24 h. Given the rapid and efficient targeting of these particles, the authors of these studies propose them as promising drug and gene delivery systems targeted to the heart.

#### Brain targeting

The studies included in this review have addressed the targeting to the brain by employing molecules that bind to the transferrin receptor, insulin receptor, or glucose transporter-1.

##### Transferrin receptor (TfR)

Transferrin (Tf) or monoclonal antibodies (mAb) targeting the transferrin receptor (TfR) have been assessed as potential targeting agents for the brain. Zhao et al. [29] functionalized liposomes with Tf to deliver pDNA encoding vascular endothelial growth factor (VEGF) to the brain of experimental stroke rats. Forty-eight hours after i.v. injection of Tf-liposomes to rats, the levels of the therapeutic protein in the brain significantly increased compared with unmodified liposomes, and they observed an attenuation of the ischemic brain injury at day 21. Another study explored the expression of an exogenous gene in the brain following non-invasive i.v. administration to rats of a pDNA encoding either luciferase or β-galactosidase, formulated within neutral immunoliposomes modified with polyethylene glycol (PEG) and conjugated with the OX26 mAb targeting the rat TfR [30, 31]. The authors observed a widespread gene expression throughout the central nervous system, encompassing neurons, choroid plexus epithelium, and the brain microvasculature. Additionally, when the immunoliposomes were conjugated with the 8D3 mAb targeting the mouse TfR and administered to mice [32], the transgene was expressed in both brain and TfR-rich peripheral tissues (liver, spleen and lungs) when employing the simian virus 40 promoter. However, when utilizing the brain-specific GFAP promoter, expression of the exogenous gene was restricted to the brain. In further studies, they evaluated the applicability of those brain-targeted liposomes for the delivery of therapeutic pDNAs in animal models of Parkinson’s disease [33–36], and type VII mucopolysaccharidosis [37].

Dual surface modified liposomes with Tf and cell-penetrating peptides (CPPs) have also been developed for delivering desired genes across the BBB in vivo. Several CPPs have been tested along with Tf as targeting ligand in liposomes [38–43], including poly-L-arginine, penetratin, vascular endothelial-cadherin-derived peptide, pentapeptide QLPVM, HIV-1 trans-activating protein (TAT), melittin, Kaposi fibroblast growth factor (kFGF), and penetration accelerating sequence–R8. Overall, biodistribution and gene expression studies in healthy and disease mouse and rat models showed that dual-ligand liposomes present a significantly higher ability to cross the BBB and to transfect brain tissue, including neurons, as compared to single-ligand liposomes with either Tf or CPPs, or plain liposomes.

##### Insulin receptor

Zhang Y et al. tested the applicability of the insulin receptor as a target to deliver exogenous genes to the brain of primates [44]. To do so, they encapsulated expression plasmids encoding luciferase or β-galactosidase within an “artificial virus” formed by a PEGylated immunoliposome conjugated to a mAb (83 − 14) targeting the human insulin receptor. The system was evaluated in vivo after i.v. administration in rhesus monkeys and the gene expression was compared to that obtained in rats with liposomes functionalized with the mAb OX26 that recognizes the rat TfR, as mentioned above. Luciferase gene expression levels in the rhesus monkey brain was 50-fold higher compared to rats. Histological analysis and confocal microscopy showed widespread neuronal expression of the β-galactosidase gene in the primate brain. As the authors discuss, the higher levels of gene expression following targeting of the insulin receptor may be attributed to the property of this receptor to internalize and translocate to the nucleus. In a subsequent study, Chu et al. [45] studied the durability of the gene expression after a single i.v. injection of pDNA encapsulated within those PEGylated immunoliposomes targeted to the human insulin receptor. They found that luciferase expression in primate brain and liver decays with a half-life (t_1/2_) of about 2 days following the administration, and detected a correlation between the rate of loss of expression of foreign genes in the primates in vivo and the degradation rate of the introduced pDNA.

##### Glucose transporter-1 (GLUT-1)

Arora et al. [46] have shown the targeted delivery of the brain-derived neurotrophic factor (BDNF) gene to the brain utilizing liposomes modified with a GLUT-1 targeting ligand (mannose) and CPPs (penetratin or rabies virus glycoprotein). The decorated liposomes exhibited remarkably elevated expression rates of BDNF in primary astrocytes and neurons when compared to unmodified liposomes. Moreover, dual-modified liposomes with mannose and CPPs demonstrated around 50% greater permeability across an in vitro BBB model. In vitro results correlated with significantly higher transport across BBB and BDNF expression, following single intravenous administration of surface modified liposomes in C57BL/6 mice, without any signs of inflammation or toxicity.

#### Liver targeting

Targeting to the liver has been mainly addressed with ligands that bind the asialoglycoprotein receptor (ASGPr) or the low density lipoprotein (LDL) receptor, although other alternatives have also been considered, including lactoferrin, natural β-sitosterol β-D-glucoside or hepatotropic viral proteins.

##### Asialoglycoprotein receptor (ASGPr)

Asialoglycoprotein receptor (ASGPR)-mediated endocytosis has been used to target genes to hepatocytes in vivo. Molecules containing exposed galactose or N-acetylgalactosamine residues, which are able to bind to the ASGPr, have been proposed as targeting moieties. Kawakami et al. [47] synthetized monosaccharide ligand-anchored cholesterol glycolipids to obtain galactosylated (Gal), mannosylated (Man) and fucosylated (Fuc) liposomes, and they compared the in vivo disposition and the pharmacokinetic profile following i.v. injection to mice. All the glycosylated liposomes were preferentially detected in the liver. However, Gal liposomes were distributed mainly in parenchymal cells (PC), corresponding to hepatocytes, while Man and Fuc liposomes were internalized by non-parenchymal cells (NPC).

Galactomannan is a polysaccharide containing galactose groups that has been used to target solid lipid nanoparticles (SLN) to the ASGPr and to deliver pDNA encoding α-Gal A to the liver [48]. The lipid-based vector did not show relevant agglutination of erythrocytes and lacked hemolytic activity in vitro, and after systemic administration to a mouse model of FD, clinically relevant α-Gal A activity levels were achieved in plasma, liver, and other organs, importantly in heart and kidneys, two of the most damaged organs in FD. The elevated enzyme activity detected in blood suggests that the enzyme could have been produced in the liver and distributed to other organs, but this point was not confirmed.

Asialofetuin (AF) has been employed by different researchers to target vectors to the ASGPr. Dasí et al. [49] covalently coupled AF to the surface of anionic and cationic liposomes to deliver the human α1-antitrypsin (hAAT) gene to mice in vivo. AF-liposomes increased the plasma levels of the hAAT and mediated long-term gene expression (> 12 months) in mice. Authors confirmed that the liver was the source of the protein. In a later work, Arangoa et al. [50] developed a cationic liposome functionalized with AF and containing protamine sulfate and a plasmid that encoded luciferase. Upon i.v. administration to mice luciferase gene expression increased by a factor of 12 in the liver compared to plain complexes, and transfection was mainly achieved in hepatocytes.

N-acetylgalactosamine (GalNAc) is another well-known ligand of the ASGPr. Prieve et al. [51] combined two types of nanoparticles in a novel hybrid mRNA delivery system targeting the liver: a GalNAc-targeted polymer micelle, and an inert lipid nanoparticle (LNP). Administration by i.v. injection of the hybrid delivery system resulted in liver-specific expression of ornithine transcarbamylase enzyme without any detectable expression in other tissues. Repeated doses led to a prolonged survival benefit in a hyperammonemic murine model of ornithine transcarbamylase deficiency.

Mukthavaram et al. [52] designed and synthesized two novel series of cationic glycolipids with cyclic and open D-galactose heads containing space arms with different lengths between the sugar and positively charged nitrogen atoms to prepare liposomes for selective gene targeting to liver mediated by the ASGPr. Authors demonstrated that cationic glycolipids with cyclic sugar-head required longer spacer arms than their acyclic sugar-head counterparts for efficient gene transfection.

The fusogenic galactose-terminated F-glycoprotein of the Sendai virus has been employed for targeted delivery of liposomes encapsulating pDNA to hepatocytes via ASGPr [53]. The liposomal system containing the human uridinediphosphoglucuronate glucuronosyltransferase-1A1 gene (hUGT1A1) was administered through i.v. route into UGT1A1-deficient hyperbilirubinemic Gunn rats (model of Crigler-Najjar syndrome type 1). Gene expression was detectable only in the liver; specifically, hUGT1A1 expression was identified in 5–10% of hepatocytes, but not in other cell types.

##### Low-density lipoprotein (LDL) receptor-related family of receptors

Apolipoprotein E (ApoE) binds with high‑affinity to several receptors such as LDL receptor. However, recombinant ApoE protein is too large (34 kDa) to be used as a ligand for pDNA lipoplexes. In a work carried out by Hattori Y et al. [54], as an alternative to recombinant ApoE, two ApoE-derived peptides, dApoE-R9 and ApoE-F-R9, were synthesized as liver-targeting moieties of cationic liposomes. These peptides include nine terminal arginine residues for interaction with pDNA. After i.v. administration to mice of liposomes functionalized with these peptides, the authors concluded that liposomes functionalized with the dApoE-R9 derivatives resulted to be the most efficient in transfecting the liver.

##### Others

Weeke-Klimp et al. [55] prepared lactoferrin (LF)-coupled liposomes to deliver pDNA specifically to hepatocytes, since hepatocytes have two major binding sites for LF. After i.v. injection to rats, about 87% of the LF-liposomes disappeared from the blood within 5 min, while 80% of untargeted liposomes were still circulating after 2 h. 52% of the LF-liposomes were taken up by hepatocytes. The targeting to hepatocytes was very efficient, but no significant transfection was observed, neither in hepatocytes nor in any other cell type in the liver or in any other organ. Authors considered that the LF–liposomes were too stable after cellular uptake and, therefore, were not able to release sufficient amounts of the plasmid from the endosomal compartment to detect transfection in vivo.

Natural β-sitosterol β-D-glucoside (Sit-G) from soybean-derived sterylglucoside has also been investigated for targeting liposomes to the liver [56]. Twenty-four hours after the systemic administration to mice, liposomes functionalized with Sit-G showed significantly and selectively higher gene expression in the liver as compared to unmodified liposomes, which exerted a higher efficacy in the lungs. However, authors highlight that it is necessary to clarify whether gene expression in the liver is selective for PCs or NPCs.

Virus like particles have been also explored as targeted delivery systems. In this regard, peptides or fragments of viruses that possess hepatotropic properties have been tested. Liposomes decorated with the preS domain of hepatitis B virus [57] were injected to immunocompromised mice via tail vein and β-galactosidase mRNA levels and enzyme activity were quantified. Gene expression with preS-liposomes took place mainly in the liver, while the expression with uncoated liposomes was mainly distributed in the lungs. A significantly higher β-galactosidase activity was found in liver relative to other organs, including lung, kidney, skin and heart 24 and 48 h after administration. Hepatitis B virus surface protein (HBsAg) has been used to develop liposomes with strict hepatotropism [58]. The biodistribution behaviour of radiactive of plain and HBsAg coated liposomes following i.v. injection to rats revealed that almost 75% of the radioactivity was recovered in the liver 4 h after injection, which was nearly three-fold greater in magnitude than the plain liposomes. Within the liver, HBsAg coated liposomal carriers were preferentially localized in the PCs.

## Discussion

Following systemic administration, the biological processes that nanoparticles undergo, conditioned by their physicochemical properties, affect their fate in the body, making the targeting to specific organs challenging. Multiple mechanisms can be utilized separately or in combination to control where in the body nanoparticles accumulate, including passive, endogenous and active targeting strategies [59, 60]. Passive targeting relies on modulating physical and chemical properties of nanoparticles, such as size, shape, charge and surface coating for nanoparticles to effectively interact with anatomy and physiology of the target organ [61]. In this regards, nanoparticles tend to distribute across various organs in a size-dependent fashion, exhibiting the highest accumulation in the liver and spleen [62, 63]. In addition, clearance of nanoparticles from the circulation often results from interactions with cells featuring the mononuclear phagocytic system, which also favors their accumulation in the spleen and liver, being cationic nanoparticles generally those that are most rapidly taken up this way [62]. Endogenous targeting is a new approach based on the plasma protein corona adsorbed to the nanoparticle surface upon contact with the blood [60, 64]. This targeting strategy involves modifying the composition of nanoparticles to promote their binding to specific plasma proteins after injection to guide them to a particular organ, promoting their uptake by specific cells within that organ [65]. In contrast, active targeting involves the use of specific ligands, antibodies, or other molecules on the surface of nanocarriers to bind to receptors or antigens that are uniquely expressed, or overexpressed, on target cells, allowing for precise delivery [66].

Our study systematically reviews molecules that have been used to target lipid-based pDNA or mRNA delivery systems intravenously to the organs therapeutically relevant in FD (heart, kidneys, brain and liver). The heterogeneous characteristics between the target tissues in FD requires tailoring the delivery system for each specific intended destination, which presents an added challenge. The brain and liver have been the primary focus of study based on the search conducted in this systematic review, whereas the reports that refer to active targeting of lipid systems to heart, kidney, endothelial and smooth muscle cells are scarce or null.

The physiological BBB represents the major obstacle for the delivery of bioactives to the brain through systemic route. In fact, currently available ERT drugs for FD are unable to cross the BBB. The use of the TfR as a target to bypass the BBB has shown promising results, with both Tf and mAb against TfR proving effective in delivering nucleic acids to the brain [29–37]. This strategy has been documented in publications prior to 2011, but the studies have not progressed beyond the preclinical phase. This may be due to two important limitations of the TfR as target: off-target effects in TfR-rich peripheral tissues, including liver, spleen and lungs, and receptor saturation. Nevertheless, in FD, which presents multisystemic affectations, the expression of the therapeutic protein in more than one target organ, for example, brain and liver, can be beneficial to address symptoms. Regarding receptor saturation, it can be overcome by using dual surface modified liposomes. This approach has enhanced the effectiveness of brain targeting by synergistically combining CPPs with ligands targeting brain receptors, such as Tf targeted to TfR [38–43] or mannose targeted to GLUT-1 receptor [46]. This promising strategy may have more possibilities of progression to clinic. In fact, brain-targeted liposomes patents have arisen derived from this combination strategy [67, 68]. In addition to the molecules identified in this review to target pDNA and mRNA to the brain, several other molecules have been used to deliver different active molecules into brain, which could be applicable to the delivery of nucleic acids. For instance, targeting to the brain has been approached by using the amyloid precursor protein (APP) [69], glutathione [70] or peptides targeted to the epidermal growth factor receptor (EGFR) [71]. Additionally naturally occurring molecules have also demonstrated brain targeting ability, such as quorum sensing peptides [72], which are peptides that bacteria use to communicate, and venom-derived peptides, including peptide apamin from bee venom [73] and chlorotoxin (CTX) from scorpion venom [74]. However, their toxicity hinders their clinical development and modifications in their molecular structure result essential to improve their biocompatibility. Furthermore, ApoE has been used to target nanoparticles to the brain owing to the expression of low-density lipoprotein (LDL) receptor related protein1 (LRP1) and very low-density lipoprotein (VLDL) receptor on brain endothelial cells [75]. The first attempts consisted on endogenous targeting mediated by modifications of nanoparticles surface (e.g. with polysorbate 80) to favor adsorption of ApoE once in the blood stream [76, 77]. Later on, in order to reduce the effect of the variability of the protein corona among individuals, ApoE and ApoE-derived peptides were covalently linked to the surface of nanoparticles for active targeting [78, 79].

Nevertheless, ApoE-mediated targeting also results in nanoparticle uptake in other tissues that express LDL receptors and are more accessible than the brain, such as the liver [79, 80]. In fact, ApoE-derived peptides have been included in this systematic review as a strategy for active targeting to liver of liposomes after i.v. administration [54]. Moreover, it has been extensively demonstrated that the physicochemical similarity of the commonly referred to as LNPs to VLDL and the propensity to adsorb ApoE in blood plasma, enhances the accumulation of this kind of lipid systems in the liver and the internalization into hepatocytes via the LDL receptor [81]. Although the reports identified here for active liver targeting highlight the extensive efforts and strategies employed to efficiently deliver therapeutic nucleic acids to hepatocytes, LNPs have emerged as the most advanced non-viral carriers for delivering nucleic acids to liver. However, the hepatocyte-targeting ability of LNPs relays on endogenous mechanisms, and for this reason, scarce studies involving LNPs have been identified in this systematic review, focused on active targeting. In this regard, LNPs lacking any active targeting ligand have been studied to deliver mRNA encoding α-Gal A to hepatocytes of Fabry mice and non-human primates after i.v. administration [82, 83]. Despite further studies with these LNPs have not been reported up to date, initial results showed the production of functional α-Gal A in the liver, which then was secreted into the circulation. Secreted α-Gal A was taken up by distal tissues such as kidney, heart, and spleen and attenuated substrate accumulation in affected tissues. These hallmarks, while valuable for liver hepatocyte applications, severely limit the use of these LNP technologies beyond the liver. Recently, a methodology termed selective organ targeting (SORT) has been developed, which enables controllable delivery of nucleic acids to target tissues [65]. SORT LNPs involve the inclusion of SORT molecules, such as charge-based lipids, that accurately tune delivery to extrahepatic tissues after i.v. administration. This strategy, added to the recognized efficacy of LNPs, represents a great advance in the development of systems targeting extrahepatic organs, and it has been included in at least two patents [84, 85]. The SORT LNPs have been developed so far to target liver, lung and spleen [86], but the rapid evolution in the development of new lipids will likely make vectorization to many other organs possible in the very near future.

The absence of in vivo studies utilizing active targeted lipid systems for pDNA or mRNA delivery to the kidney, resident smooth muscle cells or vascular endothelial cells, represents a significant gap in the current landscape of nucleic acid delivery research, not only for FD but also for other pathological conditions. Nevertheless, in vivo delivery of nucleic acids other than pDNA or mRNA, such as siRNAs or ASOs, to kidney and vascular endothelial cells with actively targeted lipid nanocarriers has been investigated, and these strategies could be applied to pDNA and mRNA delivery. For instance, liposomes functionalized with anti-Thy 1 antibody OX-7 to target the kidney [87], and with antibodies against vascular cell adhesion protein 1 (VCAM-1) for vascular endothelium targeting [88].

Active targeting of nucleic acid lipid delivery systems offer great potential for precision medicine but face several hurdles on the path to clinical translation. The key for receptor recognition and targeted delivery is the density, distribution and conformation of targeting ligands on the outer surface of the lipid carriers. However, it remains as a major challenge to precisely and controllably modulate and characterize the presentation of surface ligands, which is highly relevant for large-scale manufacturing. The main methods identified in this review for anchoring targeting molecules to the surface of lipid-based nanoparticles were: (1) one-pot assembly of all lipids and targeting ligands or targeting ligand-modified lipids, or (2) post-insertion of targeting ligands into preformed plain nanoparticles. Nevertheless, those methods are generally based on reactions that are difficult to modulate, leading to non-specific surface conjugation. High-throughput formulation screening and the stability of targeted lipid delivery systems in complex in vivo conditions, as well as for long-term storage, are two other important aspects to be taken into account to take a step forward [21]. In addition to those formulation-related factors, inefficient scaling-up methods and the lack of in vitro and in vivo correlation, a highly frequent bottleneck in nucleic acid-based therapies, represent two major limitations for clinical translation [89]. In fact, despite the wide range of organ-specific ligands that have been described, and the extensive experience with active targeting at preclinical level, endogenous targeting mechanism is taking the lead. While actively-targeted lipid-based systems for nucleic acid delivery have not been documented in clinical trials, several intravenously administered LNPs have reached the clinical evaluation with the therapeutic aim of delivering nucleic acids to liver [90]. It has been demonstrated that the liver tropism, for example in the case of Onpattro, approved in 2018 for the treatment of hereditary transthyretin-mediated amyloidosis (hATTR amyloidosis) by siRNA, is based on the endogenous targeting mediated by ApoE [91]. Nevertheless, it has to be taken into account that the complexity of biological systems might slow down the complete characterization of the in vivo protein corona behavior and, therefore, the achievement of a deep understanding of the endogenous targeting mechanisms [92].

The reliability and scientific validity of preclinical experiments in animals is crucial for the clinical translation of novel advanced therapies. It is therefore important to ensure proper design, accurate analysis, and transparent reporting to the scientific community. According to the quality assessment, the preclinical studies included in the present systematic review has shown an overall quality coefficient of nearly 0.5 over 1, which is considered to be of average quality. Furthermore, the coefficient for each individual study tends to be higher for the most recent studies, as shown in **Table S2** (Online Resource 1), indicating a raising awareness of the importance of using the ARRIVE 2.0 guidelines when reporting animal studies, which will facilitate the clinical translation of revolutionary nanomedicines.

## Conclusions

The results presented here facilitate the identification of ligands that could be used to decorate lipid-based systems carrying sequences that encode the α-Gal A for specific delivery to the organs of interest as a novel strategy to treat FD. Several molecules have been identified to target brain and liver. However, the targeting to heart, kidney, smooth muscle and endothelial cells for protein supplementation by nucleic acids has scarcely been addressed to date. Active targeting systems still require further optimization in terms of reproducibility, characterization and large-scale production to approach clinical translation, and alternative strategies such as endogenous targeting by modifying lipid composition to modulate protein corona may be more reliable in a near future.

## Electronic supplementary material

Below is the link to the electronic supplementary material.

Supplementary Material 1

## Data Availability

Not applicable.
