# Supplementary material for: Targeting strategies with lipid vectors for nucleic acid supplementation therapy in Fabry disease: a systematic review
Source: Drug Deliv Transl Res. 2024 Apr 8;14(10):2615–28. doi: 10.1007/s13346-024-01583-0 (PMC11383842; doi:10.1007/s13346-024-01583-0)
Supplement: Supplementary file 1 — Supplementary Material 1 [file 13346_2024_1583_MOESM1_ESM.docx]

**Supplementary information**

**Targeting Strategies with Lipid Vectors for Nucleic Acid Supplementation Therapy in Fabry Disease: A Systematic Review**

**Drug Delivery and Translational Research**

An Official Journal of the Controlled Release Society

Special issue: **Unlocking the Potential of Nanomedicine: Advances in Precision Targeting Strategies**

Julen Rodríguez-Castejón^1,2^, Marina Beraza-Millor^1,2^, María Ángeles Solinís^1,2^, Alicia Rodríguez-Gascón^1,2^ and Ana del Pozo-Rodríguez^1,2,*^

^1^ Pharmacokinetic, Nanotechnology and Gene Therapy Group (PharmaNanoGene), Faculty of Pharmacy, Centro de Investigación Lascaray Ikergunea, University of the Basque Country, UPV/EHU, Paseo de la Universidad 7, 01006 Vitoria-Gasteiz, Spain

^2^ Bioaraba, Microbiology, Infectious Disease, Antimicrobial Agents, and Gene Therapy, 01006 Vitoria-Gasteiz, Spain

* Correspondence: ana.delpozo@ehu.eus, tel.: +34-945-014-498

**Table S1.** Full search strategy for each individual database

| Database | Search strategy |
| --- | --- |
| PubMed | (heart[Title/Abstract] OR kidney*[Title/Abstract] OR brain[Title/Abstract] OR liver[Title/Abstract] OR muscle[Title/Abstract] OR "endothelial cell*"[Title/Abstract]) AND ("gene therap*"[Title/Abstract] OR "gene delivery"[Title/Abstract] OR "gene transfer"[Title/Abstract] OR "gene supplementation"[Title/Abstract] OR "mRNA therap*"[Title/Abstract] OR "mRNA delivery"[Title/Abstract]) AND (targeting[Title/Abstract] OR targeted[Title/Abstract] OR functionalized[Title/Abstract]) AND ("lipid nanocarrier*"[Title/Abstract] OR "lipid nanoparticle*"[Title/Abstract] OR liposome*[Title/Abstract]) AND (intravenous[Title/Abstract] OR systemic[Title/Abstract]) NOT (siRNA[Title/Abstract] OR ASO[Title/Abstract] OR "antisense oligonucleotide*"[Title/Abstract]) NOT (review[Publication Type] OR letter[Publication Type] OR editorial[Publication Type]) AND (english[Language]) |
| Web of Science (WoS) | TI=((heart OR kidney* OR brain OR liver OR muscle OR "endothelial cell*") AND ("gene therap*" OR "gene delivery" OR "gene transfer" OR "gene supplementation" OR "mRNA therap*" OR "mRNA delivery") AND (targeting OR targeted OR functionalized) AND ("lipid nanocarrier*" OR "lipid nanoparticle*" OR liposome*) AND (intravenous OR systemic) NOT (siRNA OR ASO OR "antisense oligonucleotide*")) OR AB=((heart OR kidney* OR brain OR liver OR muscle OR "endothelial cell*") AND ("gene therap*" OR "gene delivery" OR "gene transfer" OR "gene supplementation" OR "mRNA therap*" OR "mRNA delivery") AND (targeting OR targeted OR functionalized) AND ("lipid nanocarrier*" OR "lipid nanoparticle*" OR liposome*) AND (intravenous OR systemic) NOT (siRNA OR ASO OR "antisense oligonucleotide*")) NOT DT=(Review OR Letter OR Editorial Material OR Book Chapter OR Proceedings Paper) AND LA=(English) |
| Scopus | TITLE-ABS-KEY ( ( heart OR kidney* OR brain OR liver OR muscle OR "endothelial cell*" ) AND ( "gene therap*" OR "gene delivery" OR "gene transfer" OR "gene supplementation" OR "mRNA therap*" OR "mRNA delivery" ) AND ( targeting OR targeted OR functionalized ) AND ( "lipid nanocarrier*" OR "lipid nanoparticle*" OR liposome* ) AND ( intravenous OR systemic ) AND NOT ( siRNA OR ASO OR "antisense oligonucleotide*" ) ) AND NOT DOCTYPE ( re OR le OR ed OR ch OR cp ) AND LANGUAGE ( english ) |

**Table S2.** Quality assessment of studies following the ARRIVE 2.0 guidelines (Animal Research: Reporting of *In Vivo* Experiments 2.0)

|  |  | Items | | | | | | | | | | | | | | | | | | | | |  |  |
| --- | --- | --- | --- | --- | --- | --- | --- | --- | --- | --- | --- | --- | --- | --- | --- | --- | --- | --- | --- | --- | --- | --- | --- | --- |
| References | **Year** | **1** | **2** | **3** | **4** | **5** | **6** | **7** | **8** | **9** | **10** | **11** | **12** | **13** | **14** | **15** | **16** | **17** | **18** | **19** | **20** | **21** | **Total score** | **Coefficient** |
| Kawakami et al. [1] | 2000 | 2 | 0 | 0 | 0 | 0 | 1 | 0 | 2 | 1 | 2 | 1 | 1 | 2 | 0 | 0 | 1 | 1 | 0 | 0 | 0 | 1 | **15** | **0.36** |
| Shi et al. [2] | 2000 | 2 | 0 | 1 | 0 | 0 | 2 | 0 | 1 | 1 | 2 | 0 | 1 | 2 | 0 | 0 | 1 | 1 | 2 | 0 | 0 | 0 | **16** | **0.38** |
| Shi et al. [3] | 2001 | 2 | 1 | 1 | 0 | 0 | 2 | 0 | 2 | 1 | 2 | 2 | 2 | 2 | 0 | 0 | 1 | 1 | 2 | 0 | 0 | 2 | **23** | **0.55** |
| Hwang et al. [4] | 2001 | 2 | 1 | 0 | 0 | 0 | 2 | 1 | 2 | 2 | 2 | 2 | 1 | 2 | 0 | 0 | 1 | 1 | 0 | 0 | 0 | 1 | **20** | **0.48** |
| Shi et al. [5] | 2001 | 2 | 0 | 0 | 0 | 0 | 2 | 0 | 0 | 1 | 0 | 1 | 2 | 0 | 0 | 1 | 1 | 0 | 0 | 0 | 0 | 0 | **10** | **0.24** |
| Dasí et al. [6] | 2001 | 2 | 1 | 0 | 0 | 0 | 2 | 0 | 0 | 1 | 0 | 0 | 1 | 2 | 0 | 0 | 0 | 1 | 0 | 0 | 0 | 1 | **11** | **0.26** |
| Zhang Y et al. [7] | 2003 | 2 | 1 | 0 | 0 | 0 | 1 | 0 | 2 | 2 | 2 | 2 | 1 | 2 | 0 | 0 | 1 | 1 | 0 | 0 | 0 | 1 | **18** | **0.43** |
| Zhang Y et al. [8] | 2003 | 1 | 0 | 0 | 0 | 0 | 1 | 0 | 1 | 2 | 2 | 2 | 1 | 2 | 0 | 0 | 1 | 1 | 0 | 0 | 0 | 2 | **16** | **0.38** |
| Arangoa et al. [9] | 2003 | 2 | 1 | 0 | 0 | 0 | 2 | 0 | 2 | 1 | 2 | 2 | 1 | 2 | 2 | 0 | 0 | 1 | 0 | 0 | 0 | 1 | **19** | **0.45** |
| Zhang Y et al. [10] | 2004 | 2 | 1 | 0 | 0 | 0 | 2 | 1 | 2 | 1 | 2 | 0 | 2 | 2 | 0 | 0 | 1 | 1 | 2 | 0 | 0 | 1 | **20** | **0.48** |
| Khatri et al. [11] | 2005 | 2 | 1 | 0 | 0 | 0 | 2 | 0 | 0 | 2 | 2 | 0 | 1 | 1 | 2 | 2 | 1 | 1 | 0 | 0 | 0 | 1 | **18** | **0.43** |
| Chu et al. [12] | 2006 | 2 | 1 | 1 | 0 | 0 | 1 | 1 | 2 | 2 | 2 | 2 | 1 | 2 | 0 | 0 | 1 | 1 | 0 | 0 | 0 | 1 | **20** | **0.48** |
| Weeke-Klimp et al. [13] | 2007 | 2 | 0 | 0 | 0 | 0 | 2 | 0 | 2 | 2 | 2 | 0 | 1 | 2 | 2 | 2 | 1 | 1 | 0 | 0 | 0 | 1 | **20** | **0.48** |
| Zhang H et al. [14] | 2008 | 2 | 1 | 1 | 0 | 0 | 2 | 1 | 2 | 2 | 2 | 0 | 1 | 0 | 2 | 2 | 2 | 1 | 0 | 0 | 0 | 1 | **22** | **0.52** |
| Wang Z et al. [15] | 2008 | 2 | 0 | 0 | 0 | 0 | 2 | 0 | 0 | 2 | 2 | 2 | 1 | 2 | 0 | 0 | 0 | 1 | 0 | 0 | 0 | 2 | **16** | **0.38** |
| Xia et al. [16] | 2008 | 2 | 0 | 0 | 0 | 0 | 1 | 1 | 2 | 2 | 2 | 2 | 1 | 2 | 2 | 0 | 1 | 1 | 2 | 0 | 0 | 1 | **22** | **0.52** |
| Zhang et al. [17] | 2008 | 2 | 0 | 0 | 0 | 0 | 2 | 1 | 2 | 1 | 2 | 0 | 1 | 2 | 2 | 0 | 0 | 1 | 0 | 0 | 0 | 0 | **16** | **0.38** |
| Mukthavaram et al. [18] | 2009 | 2 | 1 | 1 | 0 | 0 | 2 | 0 | 2 | 1 | 2 | 2 | 1 | 2 | 2 | 0 | 0 | 1 | 0 | 0 | 0 | 1 | **20** | **0.48** |
| Wang X et al. [19] | 2009 | 2 | 0 | 0 | 0 | 0 | 2 | 0 | 1 | 2 | 2 | 2 | 2 | 2 | 2 | 2 | 0 | 1 | 0 | 0 | 0 | 2 | **22** | **0.52** |
| Zhang Y et al. [20] | 2009 | 2 | 1 | 0 | 0 | 0 | 2 | 0 | 2 | 1 | 2 | 2 | 0 | 0 | 2 | 0 | 1 | 1 | 0 | 0 | 0 | 1 | **17** | **0.40** |
| Zhao et al. [21] | 2011 | 2 | 1 | 1 | 0 | 0 | 2 | 1 | 2 | 2 | 2 | 2 | 1 | 2 | 0 | 2 | 1 | 1 | 0 | 0 | 0 | 2 | **24** | **0.57** |
| Zhang H et al. [22] | 2012 | 2 | 1 | 0 | 0 | 0 | 2 | 1 | 2 | 1 | 2 | 2 | 2 | 2 | 2 | 0 | 0 | 1 | 0 | 0 | 0 | 1 | **21** | **0.50** |
| Sharma et al. [23] | 2013 | 2 | 1 | 1 | 0 | 0 | 2 | 1 | 1 | 2 | 2 | 2 | 1 | 2 | 2 | 2 | 0 | 1 | 0 | 0 | 0 | 1 | **23** | **0.55** |
| Prieve et al. [24] | 2018 | 2 | 2 | 1 | 1 | 0 | 2 | 2 | 2 | 1 | 2 | 2 | 2 | 2 | 2 | 0 | 0 | 1 | 2 | 0 | 2 | 2 | **30** | **0.71** |
| Dos Santos Rodrigues et al. [25] | 2018 | 2 | 1 | 0 | 0 | 0 | 2 | 1 | 0 | 2 | 2 | 0 | 1 | 2 | 2 | 2 | 0 | 2 | 0 | 0 | 0 | 1 | **20** | **0.48** |
| Dos Santos Rodrigues et al. [26] | 2019 | 2 | 1 | 1 | 0 | 0 | 2 | 0 | 1 | 2 | 2 | 1 | 1 | 1 | 2 | 2 | 0 | 2 | 0 | 0 | 0 | 2 | **22** | **0.52** |
| Hattori et al. [27] | 2019 | 2 | 1 | 1 | 0 | 0 | 2 | 1 | 2 | 2 | 2 | 0 | 1 | 2 | 2 | 2 | 0 | 1 | 2 | 0 | 2 | 2 | **27** | **0.64** |
| Dos Santos Rodrigues et al. [28] | 2019 | 2 | 1 | 0 | 0 | 0 | 2 | 1 | 2 | 1 | 2 | 0 | 1 | 2 | 2 | 2 | 0 | 1 | 0 | 0 | 0 | 2 | **21** | **0.50** |
| Dos Santos Rodrigues et al. [29] | 2020 | 2 | 1 | 1 | 0 | 0 | 1 | 1 | 2 | 2 | 2 | 2 | 1 | 2 | 2 | 2 | 0 | 1 | 0 | 0 | 0 | 1 | **23** | **0.55** |
| Arora et al. [30] | 2020 | 0 | 1 | 1 | 0 | 0 | 1 | 1 | 2 | 2 | 2 | 2 | 1 | 2 | 2 | 2 | 0 | 1 | 0 | 2 | 2 | 2 | **26** | **0.62** |
| Dos Santos Rodrigues et al. [31] | 2020 | 2 | 0 | 0 | 0 | 0 | 2 | 0 | 2 | 2 | 2 | 2 | 2 | 2 | 2 | 2 | 0 | 1 | 0 | 0 | 0 | 2 | **23** | **0.55** |
| Rodríguez-Castejón et al. [32] | 2022 | 2 | 1 | 1 | 0 | 0 | 1 | 2 | 2 | 2 | 1 | 2 | 2 | 2 | 2 | 2 | 1 | 1 | 0 | 0 | 0 | 2 | **26** | **0.62** |

**Items:** (1) study design, (2) sample size, (3) inclusion and exclusion criteria, (4) randomisation, (5) blinding/masking, (6) outcome measures, (7) statistical methods, (8) experimental animals, (9) experimental procedures, (10) results, (11) abstract, (12) background, (13) objectives, (14) ethical statement, (15) housing and husbandry, (16) animal care and monitoring, (17) interpretation/scientific implications, (18) generalisability/translation, (19) protocol registration, (20) data access, (21) declaration of interests. **Scores:** 0: not reported; 1: unclear; 2: reported.

**Table S3**. Summary of data extracted from included studies

| Target organ | Target receptor | Targeting ligand  (type of moiety) | Delivery system | Cargo | Anchoring method | Reference |
| --- | --- | --- | --- | --- | --- | --- |
| Heart | Cysteine-rich protein-2 (CRIP-2) | CRPPR  (peptide) | Liposomes | Fluorescent dye | One-pot assembly | [14, 22] |
| Brain | Transferrin receptor | Transferrin (glycoprotein) | Liposomes | pDNA | Post-insertion | [21] |
|  |  | OX26 and 8D3  (mAb against rat and mouse TfR, respectively) | Liposomes | pDNA | Post-insertion | [2, 3, 5, 7, 10, 16, 17, 20] |
|  |  | Transferrin (glycoprotein)  + CPPs | Liposomes | pDNA | One-pot assembly of CPP + post-insertion of transferrin | [23, 25, 26, 28, 29, 31] |
|  | Human insulin receptor | 83-14  (mAb) | Liposomes | pDNA | Post-insertion | [8, 12] |
|  | Glucose transporter-1 | Mannose (monosaccharide)  + CPPs | Liposomes | pDNA | One-pot assembly | [30] |
| Liver | ASGPr | Galactose  (monosaccharides) | Liposomes | Unloaded system | One-pot assembly | [1] |
|  |  | Galactomannan  (polysaccharide) | Solid lipid nanoparticles | pDNA | Post-insertion | [32] |
|  |  | Asialofetuin  (glycoprotein) | Liposomes | pDNA | Post-insertion | [6, 9] |
|  |  | N-acetylgalactosamine - GalNAc  (monosaccharide) | Hybrid lipid-polymer system | mRNA | Post-insertion | [24] |
|  |  | Cationic glycolipids with cyclic and open D-galactose heads (glycolipids) | Liposomes | pDNA | One-pot assembly | [18] |
|  |  | Fusogenic galactose-terminated F-glycoprotein of the Sendai virus (glycoprotein) | Liposomes | pDNA | One-pot assembly | [19] |
|  | LDL receptor | ApoE-derived peptides, dApoE-R9 and ApoE-F-R9 (peptides) | Liposomes | pDNA | Post-insertion | [27] |
|  | Others | Lactoferrin (glycoprotein) | Liposomes | pDNA | Post-insertion | [13] |
|  |  | β-sitosterol β-D-glucoside  (glycolipid) | Liposomes | pDNA | One-pot assembly | [4] |
|  |  | preS domain of HBV  (peptide) | Liposomes | pDNA | Post-insertion | [15] |
|  |  | HBV surface protein (protein) | Liposomes | Unloaded system | Post-insertion | [11] |

ApoE: Apolipoprotein E. ASGPr: asialoglycoprotein receptor. CPP: cell-penetrating peptide. HBV: hepatitis B virus LDL: low-density lipoprotein. mAb: monoclonal antibody. mRNA: messenger RNA. pDNA: plasmid DNA. TfR: transferrin receptor. Cell penetrating peptides (CPP) comprise poly-L-arginine, penetratin, vascular endothelial-cadherin-derived peptide, pentapeptide QLPVM, HIV-1 trans-activating protein (TAT), melittin, Kaposi fibroblast growth factor (kFGF), penetration accelerating sequence–R8 or rabies virus glycoprotein.

**References**

1. Kawakami S, Wong J, Sato A, Hattori Y, Yamashita F, Hashida M. Biodistribution characteristics of mannosylated, fucosylated, and galactosylated liposomes in mice. Biochim. Biophys. Acta – Gen. Subj. 2000;1524(2–3):258–65. https://doi.org/10.1016/S0304-4165(00)00163-X
2. Shi N, Pardridge WM. Noninvasive gene targeting to the brain. Proc. Natl. Acad. Sci. U.S.A. 2000;97(13):7567–72. https://doi.org/10.1073/pnas.130187497
3. Shi N, Zhang Y, Zhu C, Boado RJ, Pardridge WM. Brain-specific expression of an exogenous gene after i.v. administration. Proc. Natl. Acad. Sci. U.S.A. 2001;98(22):12754–9. https://doi.org/10.1073/pnas.221450098
4. Hwang SH, Hayashi K, Takayama K, Maitani Y. Liver-targeted gene transfer into a human hepatoblastoma cell line and in vivo by sterylglucoside-containing cationic liposomes. Gene Ther. 2001;8(16):1276–80. https://doi.org/10.1038/sj.gt.3301510
5. Shi N, Boado RJ, Pardridge WM. Receptor-mediated gene targeting to tissues in vivo following intravenous administration of pegylated immunoliposomes. Pharm. Res. 2001;18(8):1091–5. https://doi.org/10.1023/A:1010910523202
6. Dasí F, Benet M, Crespo J, Crespo A, Aliño SF. Asialofetuin liposome-mediated human α1-antitrypsin gene transfer in vivo results in stationary long-term gene expression. J. Mol. Med. 2001;79(4):205–12. https://doi.org/10.1007/s001090000185
7. Zhang Y, Calon F, Zhu C, Boado RJ, Pardridge WM. Intravenous nonviral gene therapy causes normalization of striatal tyrosine hydroxylase and reversal of motor impairment in experimental parkinsonism. Hum. Gene. Ther. 2003;14(1):1–12. https://doi.org/10.1089/10430340360464660
8. Zhang Y, Schlachetzki F, Pardridge WM. Global non-viral gene transfer to the promate brain following intravenous administration. Mol. Ther. 2003;7(1):11–8. https://doi.org/10.1016/S1525-0016(02)00018-7
9. Arangoa MA, Düzgüneş N, Tros de Ilarduya C. Increased receptor-mediated gene delivery to the liver by protamine-enhanced-asialofetuin-lipoplexes. Gene Ther. 2003;10(1):5–14. https://doi.org/10.1038/sj.gt.3301840
10. Zhang Y, Schlachetzki F, Zhang YF, Boado RJ, Pardridge WM. Normalization of striatal tyrosine hydroxylase and reversal of motor impairment in experimental parkinsonism with intravenous nonviral gene therapy and a brain-specific promoter. Hum. Gene Ther. 2004;15(4):339–50. https://doi.org/10.1089/104303404322959498
11. Khatri K, Rawat A, Mahor S, Gupta PN, Vyas SP. Hepatitis B surface protein docked vesicular carrier for site specific delivery to liver. J. Drug Target. 2005;13(6):359–66. https://doi.org/10.1080/10611860500230294
12. Chu C, Zhang Y, Boado RJ, Pardridge WM. Decline in exogenous gene expression in primate brain following intravenous administration is due to plasmid degradation. Pharm. Res. 2006;23(7):1586–90. https://doi.org/10.1007/s11095-006-0274-x
13. Weeke-Klimp AH, Bartsch M, Morselt HWM, Van Veen-Hof I, Meijer DKF, Scherphof GL, et al. Targeting of stabilized plasmid lipid particles to hepatocytes in vivo by means of coupled lactoferrin. J. Drug Target. 2007;15(9):585–94. https://doi.org/10.1080/10611860701502889
14. Zhang H, Kusunose J, Kheirolomoom A, Seo JW, Qi J, Watson KD, et al. Dynamic imaging of arginine-rich heart-targeted vehicles in a mouse model. Biomaterials. 2008;29(12):1976–88. https://doi.org/10.1016/j.biomaterials.2007.12.033
15. Wang Z, Yuan Z, Jin L. Gene delivery into hepatocytes with the PreS/liposome/DNA system. Biotechnol. J. 2008;3(9–10):1286–95. https://doi.org/10.1002/biot.200800125
16. Xia CF, Boado RJ, Zhang Y, Chu C, Pardridge WM. Intravenous glial-derived neurotrophic factor gene therapy of experimental Parkinson’s disease with Trojan horse liposomes and a tyrosine hydroxylase promoter. J. Gene Med. 2008;10(3):306–15. https://doi.org/10.1002/jgm.1152
17. Zhang Y, Wang Y, Boado RJ, Pardridge WM. Lysosomal enzyme replacement of the brain with intravenous non-viral gene transfer. Pharm. Res. 2008;25(2):400–6. https://doi.org/10.1007/s11095-007-9357-6
18. Mukthavaram R, Marepally S, Venkata MY, Vegi GN, Sistla R, Chaudhuri A. Cationic glycolipids with cyclic and open galactose head groups for the selective targeting of genes to mouse liver. Biomaterials. 2009;30(12):2369–84. https://doi.org/10.1016/j.biomaterials.2008.12.074
19. Wang X, Sarkar DP, Mani P, Steer CJ, Chen Y, Guha C, et al. Long-term reduction of jaundice in Gunn rats by nonviral liver-targeted delivery of Sleeping Beauty transposon. Hepatology. 2009;50(3):815–24. https://doi.org/10.1002/hep.23060
20. Zhang Y, Pardridge WM. Near complete rescue of experimental parkinson’s disease with intravenous, non-viral GDNF gene therapy. Pharm. Res. 2009;26(5):1059–63. https://doi.org/10.1007/s11095-008-9815-9
21. Zhao H, Bao XJ, Wang RZ, Li GL, Gao J, Ma SH, et al. Postacute ischemia vascular endothelial growth factor transfer by transferrin-targeted liposomes attenuates ischemic brain injury after experimental stroke in rats. Hum. Gene Ther. 2011;22(2):207–15. https://doi.org/10.1089/hum.2010.111
22. Zhang H, Li N, Sirish P, Mahakian L, Ingham E, Curry FR, et al. The cargo of CRPPR-conjugated liposomes crosses the intact murine cardiac endothelium. J. Control Release. 2012;163(1):10–7. https://doi.org/10.1016/j.jconrel.2012.06.038
23. Sharma G, Modgil A, Layek B, Arora K, Sun C, Law B, et al. Cell penetrating peptide tethered bi-ligand liposomes for delivery to brain in vivo: Biodistribution and transfection. J. Control Release. 2013;167(1):1–10. https://doi.org/10.1016/j.jconrel.2013.01.016
24. Prieve MG, Harvie P, Monahan SD, Roy D, Li AG, Blevins TL, et al. Targeted mRNA Therapy for Ornithine Transcarbamylase Deficiency. Mol. Ther. 2018;26(3):801–13. https://doi.org/10.1016/j.ymthe.2017.12.024
25. dos Santos Rodrigues B, Oue H, Banerjee A, Kanekiyo T, Singh J. Dual functionalized liposome-mediated gene delivery across triple co-culture blood brain barrier model and specific in vivo neuronal transfection. J. Control Release. 2018;286:264–78. https://doi.org/10.1016/j.jconrel.2018.07.043
26. dos Santos Rodrigues B, Kanekiyo T, Singh J. ApoE-2 Brain-Targeted Gene Therapy Through Transferrin and Penetratin Tagged Liposomal Nanoparticles. Pharm. Res. 2019;36(11):161. https://doi.org/10.1007/s11095-019-2691-7
27. Hattori Y, Nakagawa Y, Onishi H. Gene delivery into hepatic cells with ternary complexes of plasmid DNA, cationic liposomes and apolipoprotein E‑derived peptide. Exp. Ther. Med. 2019;18(4):2628–38. https://doi.org/10.3892/etm.2019.7863
28. dos Santos Rodrigues B, Lakkadwala S, Kanekiyo T, Singh J. Development and screening of brain-targeted lipid-based nanoparticles with enhanced cell penetration and gene delivery properties. Int. J. Nanomedicine. 2019;14:6497–517. https://doi.org/10.2147/IJN.S215941
29. dos Santos Rodrigues B, Lakkadwala S, Kanekiyo T, Singh J. Dual-modified liposome for targeted and enhanced gene delivery into mice brain. J. Pharmacol. Exp. Ther. 2020;374(3):354–65. https://doi.org/10.1124/jpet.119.264127
30. Arora S, Sharma D, Singh J. GLUT-1: An Effective Target to Deliver Brain-Derived Neurotrophic Factor Gene across the Blood Brain Barrier. ACS Chem. Neurosci. 2020;11:1620–33. https://doi.org/10.1021/acschemneuro.0c00076
31. dos Santos Rodrigues B, Kanekiyo T, Singh J. Nerve Growth Factor Gene Delivery across the Blood-Brain Barrier to Reduce Beta Amyloid Accumulation in AD Mice. Mol. Pharm. 2020;17(6):2054–63. https://doi.org/10.1021/acs.molpharmaceut.0c00218
32. Rodríguez-Castejón J, Gómez-Aguado I, Beraza-Millor M, Solinís MÁ, del Pozo-Rodríguez A, Rodríguez-Gascón A. Galactomannan-Decorated Lipidic Nanocarrier for Gene Supplementation Therapy in Fabry Disease. Nanomaterials. 2022;12(14):2339. https://doi.org/10.3390/nano12142339
